# The Effect of Acute Aerobic Exercise on Divergent and Convergent Thinking and Its Influence by Mood

**DOI:** 10.3390/brainsci11050546

**Published:** 2021-04-27

**Authors:** Kohei Aga, Masato Inamura, Chong Chen, Kosuke Hagiwara, Rikuto Yamashita, Masako Hirotsu, Tomoe Seki, Akiyo Takao, Yuko Fujii, Toshio Matsubara, Shin Nakagawa

**Affiliations:** Division of Neuropsychiatry, Department of Neuroscience, Yamaguchi University Graduate School of Medicine, Ube, Yamaguchi 755-8505, Japan; i001eb@yamaguchi-u.ac.jp (K.A.); g009eb@yamaguchi-u.ac.jp (M.I.); khagi@yamaguchi-u.ac.jp (K.H.); i096eb@yamaguchi-u.ac.jp (R.Y.); hirotsu@yamaguchi-u.ac.jp (M.H.); tseki@yamaguchi-u.ac.jp (T.S.); v053eb@yamaguchi-u.ac.jp (A.T.); yuko-f@yamaguchi-u.ac.jp (Y.F.); t-matsu@yamaguchi-u.ac.jp (T.M.); snakaga@yamaguchi-u.ac.jp (S.N.)

**Keywords:** aerobic exercise, creativity, convergent thinking, divergent thinking, flexibility, insight problem-solving, cognitive functions, mood, vigor, pleasure

## Abstract

Abundant evidence shows that various forms of physical exercise, even conducted briefly, may improve cognitive functions. However, the effect of physical exercise on creative thinking remains under-investigated, and the role of mood in this effect remains unclear. In the present study, we set out to investigate the effect of an acute bout of aerobic exercise on divergent and convergent thinking and whether this effect depends on the post-exercise mood. Forty healthy young adults were randomly assigned to receive a 15-min exercise or control intervention, before and after which they conducted an alternate use test measuring divergent thinking and an insight problem-solving task measuring convergent thinking. It was found that exercise enhanced divergent thinking in that it increased flexibility and fluency. Importantly, these effects were not mediated by the post-exercise mood in terms of pleasure and vigor. In contrast, the effect on convergent thinking depended on subjects’ mood after exercise: subjects reporting high vigor tended to solve more insight problems that were unsolved previously, while those reporting low vigor became less capable of solving previously unsolved problems. These findings suggest that aerobic exercise may affect both divergent and convergent thinking, with the former being mood-independent and the latter mood-dependent. If these findings can be replicated with more rigorous studies, engaging in a bout of mood, particularly vigor-enhancing aerobic exercise, may be considered a useful strategy for gaining insights into previously unsolved problems.

## 1. Introduction

A growing body of research has investigated the positive impact of a single bout of physical exercise on cognitive functions (for a meta-analysis, [1,2,3,4,5]; for a narrative review, [6,7,8]). For instance, a single bout of aerobic exercise may improve attention and executive functions [2,4,5], boost the speed of information processing [3], and enhance memory storage and retrieval [1]. Several neurobiological mechanisms have been proposed to explain these cognitive benefits, including increased production or release of lactate, cortisol, neurotrophins (e.g., BDNF, IGF-1), and neurotransmitters (e.g., serotonin, dopamine, endocannabinoids) as well as functional hemodynamic brain changes (particularly in the prefrontal cortex) following exercise [7,9,10,11,12]. Despite these fruitful findings, relatively little is known about the effects of physical exercise on creativity or creative thinking (for a brief literature review, see below).

It has been generally considered that creative thinking comprises two fundamental processes: divergent thinking, which involves stretching beyond existing ideas to create multiple, novel ones, and convergent thinking, which involves narrowing down and approaching a single correct, task-appropriate solution [13]. Performance on tests of divergent and convergent think has been reported to predict real-life creative potential, achievement, and creativity evaluated by others [14,15,16,17]. Since creative thinking is the key to invention and innovation and is indispensable for the progress of science, technology, business and management, education, art, and society as a whole [18], the search for strategies to enhance creative thinking may have great social significance.

We identified six papers [19,20,21,22,23,24] investigating the after-effects of acute physical exercise on creative thinking (i.e., creativity post-exercise) that included a control group or condition and with the full-text available (see Table S1, the literature search strategy is described in the footnote of Table S1). All of the studies evaluated divergent thinking with the original or adapted versions of the alternate uses test (AUT, [25,26]). In this test, subjects are asked to write down as many as possible uncommon, original uses of common objects, such as “bricks” and “tin cans”. The number of generated uses (i.e., fluency), the number of different conceptual categories the alternative uses are from (i.e., flexibility), and the rareness of the generated uses (i.e., originality) are commonly used as indicators of creativity. In contrast, sometimes other indicators, such as the number of details in a given response (i.e., elaboration), have also been used [27,28]. Two of the studies [21,23] also investigated the impact of exercise on convergent thinking, one using the remote associates test (RAT, [29]) and the other its adapted version the compound remote associates test (CRA, [30]). In these tests, subjects must think of a single word associated with each of three seemingly unrelated words. For instance, for the three words, “time”, “hair”, and “stretch”, the associate might be “long”; for the three words “cottage”, “swiss”, and “cake”, the compound associate might be “cheese”.

To summarize the findings, in brief, the effect of an acute bout of aerobic exercise on divergent and convergent thinking is inconsistent. Either no effect or an enhancing or impairing effect on some measures has been reported. Based on the identified studies, one trend might be speculated that exercise at too low intensity may not reliably improve divergent and convergent thinking [21,22,23]. For instance, cycling [21] or walking [23] at very light-to-light intensity (estimated based on age-estimated maximal heart rate or HR_max_, [31,32]) failed to affect fluency, flexibility, originality, or elaboration of divergent thinking; nor did they affect the CRA or RAT performance. In contrast, a 4-min walk on a treadmill or outdoors (considered very light in intensity) increased the originality of divergent thinking compared to sitting, without affecting the CRA performance [22]. On the other hand, exercise at too high intensity, for instance, cycling with maximal effort [21], may reduce the flexibility of divergent thinking and impair the RAT performance (the latter in subjects without exercise habit).

Insights from the neurobiological literature suggest that an important factor that may provide a parsimonious explanation to the above inconsistent results is mood. Since exercise influences mood and cognition through similar neurobiological mechanisms, for instance, via neurotransmitters, such as serotonin, dopamine, and endocannabinoids [7], one may expect an association between the creativity and mood effects of exercise. Thus, it is possible that two previous studies failed to find the creativity-enhancing effect because their exercise interventions using cycling [21] or walking [23] at very light to light intensity failed to change subjects’ moods. It is also possible that cycling with maximal effort impaired divergent and convergent thinking [21] because it caused exhaustion and central fatigue. Unfortunately, these studies did not examine this potential association, although an earlier study [19] reported that the creativity effect of approximately 20 min of aerobic workout or dance was independent of the mood effect. More research is required to confirm the association between the creativity and mood effects of exercise.

Furthermore, regarding the validity of the tests of divergent and convergent thinking that have been employed, the AUT has been found to reflect cognitive flexibility and suggested to be a validated measure of divergent thinking [33]. However, the RAT measuring convergent thinking has been criticized for having low validity due to its high dependence on verbal abilities (e.g., [34]). Thus, other more appropriate measures of convergent thinking or creative problem-solving are required for further research and confirmation of the effect of the exercise.

In the present study, we aimed to address these unsolved issues and test our speculations with a randomized controlled trial. We employed a 15-min automated exercise intervention program expected to be primarily moderate in intensity, the workload of which was greater than normal walking or cycling but lighter than cycling with maximal effort (as employed by Colzato et al. [21]). We predicted that exercise at this intensity might improve the participants’ mood without causing central fatigue. Importantly, immediately following the exercise and control intervention, we measured subjects’ mood at the moment using a visual analog scale in terms of pleasure, relaxation, and vigor. We predicted that the creativity effect of exercise might (at least partially) depend on its mood effect. Finally, we used two sets of creative problem-solving questions that require logical and visuospatial rather than verbal abilities to evaluate convergent thinking.

## 2. Materials and Methods

### 2.1. Participants

The study was approved by the Institutional Review Board of Yamaguchi University Hospital and preregistered on the University hospital Medical Information Network Clinical Trial Registry (UMIN-CTR, register ID: UMIN000041122). The study was a randomized controlled trial, with a between-subjects pre-test–post-test comparison design (see design and procedure below). Forty healthy subjects (all undergraduates, 11 females, 29 males, age: 22.98 ± 1.95 years) were recruited via posters placed on campus and department homepage and through word-of-mouth. This sample size was estimated with a priori power analysis based on data from Oppezzo and Schwartz [22]. With inputs of d = 0.999 (estimated from experiment 3, walk–sit vs. sit–sit), alpha = 0.05, and power of 0.8, 17 subjects per group were required, and we recruited 20 subjects per group.

The study was carried out following the latest version of the Declaration of Helsinki, and all subjects agreed to participate in this study and provided written informed consent after receiving a detailed explanation of the study. The inclusion criteria were being 20–29 years old at the time of the visit. The exclusion criteria were (1) having a history of diseases that greatly affect cardiopulmonary functions, such as chronic heart failure, (2) currently suffering from a mental illness or undergoing medical examination, (3) being a staff of our department, who receives the personnel evaluation directly by the principal investigator of this study, (4) being judged to be unsuitable for this study (e.g., bodyweight exceeding the applicable weight of the exercise bike). No participant was excluded owing to meeting any of the exclusion criteria.

### 2.2. Design and Procedure

Subjects were informed beforehand to (1) get enough sleep on the previous night, (2) refrain from drinking coffee and energy drinks, from smoking, and from engaging in intensive physical activities during the two hours before visiting the laboratory, and (3), contact the staff and reschedule the experiment if they were sick or did not feel well on the experimental day. On the experimental day, subjects first filled out a form answering their age, gender, history of smoking and alcohol drinking, education, and questions to confirm whether they adhered to the above instructions. Subjects also filled out the International physical activity questionnaire (IPAQ, [35]) indicating their level of physical activity during the past seven days and the positive and negative affect schedule (PANAS, [36]) indicating their current mood. They then completed bodyweight tests, height (for the calculation of BMI) and grip strength (Lafayette hydraulic hand dynamometer model J00105, the average of the two hands was used).

**Figure 1:**
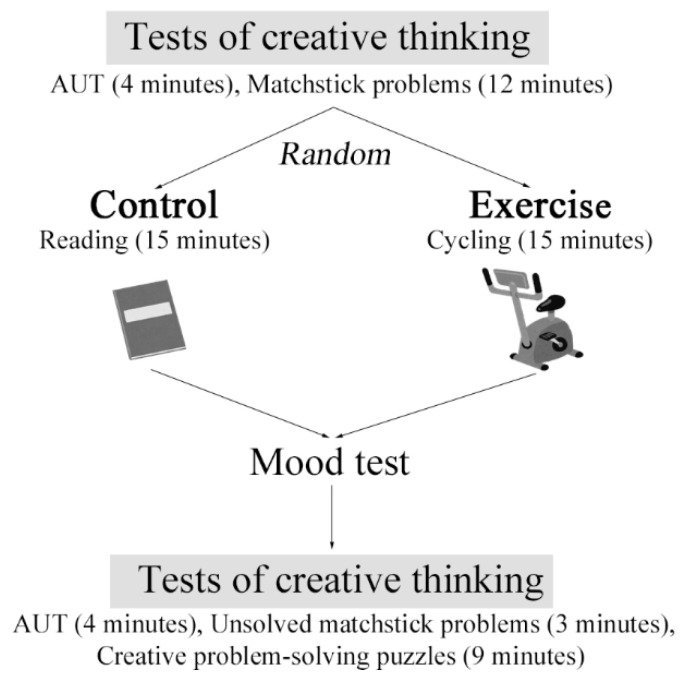
Schematic illustration of the study design.

In this study, we used a between-subjects pre-test–post-test comparison design (Figure 1). Although a within-subjects crossover pre-test–post-test design may be more rigorous [8], the nature of the creative thinking tests precluded us from using this design. Specifically, for a within-subjects crossover pre-test–post-test design, participants must effectively perform the creative thinking tests four separate times. A critical characteristic of insight problem-solving tests for the evaluation of convergent thinking is that once subjects achieve insight into a problem, they will easily solve similar problems. As the available validated insight problems are limited at the moment, it is hard or almost impossible to appropriately schedule four assessments.

Here, forty subjects were randomly assigned to receive a 15-min exercise or control intervention, before and after which they conducted tests of creative thinking that comprised a divergent thinking test and a convergent thinking test in a counterbalanced order. The statistical comparison revealed that subjects assigned to the two groups did not differ in their gender distribution, smoking and alcohol drinking habits, total metabolic equivalents (METs) of physical activity, BMI, grip strength, and positive and negative affect. The exercise group, however, had an older age than the control group (23.60 ± 2.14 vs. 22.35 ± 1.57 years, *t*(38) = 2.110, *p* = 0.041). Therefore, in our statistical analysis, we included age as a covariate.

Immediately following the intervention and before the second test of creative thinking, subjects were instructed to indicate their mood at the moment in terms of pleasure, relaxation, and vigor using a visual analog scale. This mood test was designed based on the valence-arousal two-dimensional affect grid [37]. The mood test generally took less than 30 s and immediately following the mood test, subjects conducted the second test of creative thinking. After the second test of creative thinking, subjects also filled out a set of questionnaires (measuring psychological stress, depressive and anxious symptoms, etc.) and cognitive tests (measuring decision-making and working memory), the results of which were not analyzed here.

### 2.3. Intervention

For the exercise intervention, we used the automated physical test program built in an exercise bike (Fukuda Denshi Wellbike BE-260). The program lasted 15 min and consisted of 10 min of physical test and 5 min of cooldown. Subjects were asked to sit quietly on the bike during the first minute to measure their resting state pulse rate and to start pedaling from the second minute at a pace of 50 rpm. The workload increased at 4 and 7 min after the start of the program according to the pulse rate of the subjects at the moment. For males, the workload increased to 12–25 N⋅m after 4 min and 15–35 N⋅m after 7 min, depending on the pulse rate of the subjects. For females, the workload increased to 8–15 N⋅m after 4 min and 11–25 N⋅m after 7 min, depending on the pulse rate of the subjects. Following the 10 min physical text, subjects performed a 5-min cooldown, pedaling at a self-selected pace.

Under this program, the heart rates of subjects are expected to rise to around 110–115 bpm after the first three minutes of pedaling (1–4 min), 123–135 bpm after the second three minutes of pedaling (4–7 min), and then maximally around 160 bpm after the last three minutes of pedaling (7–10 min). Since exercise intensity at <57% HR_max_ is considered very light, 57–63% HR_max_ light, 64–76% HR_max_ moderate, and 77–95% HR_max_ vigorous [31], the intensity of the current program is predicted to be very light to light during the first three minutes, light to moderate during the second three minutes, and moderate to vigorous during the third minute (assuming a mean age of 25 years and using the formula 220 minus mean age for the estimation of HR_max_, [32]). As a whole, we expected that the intensity and workload of the exercise program fall between the two interventions employed investigated by Colzato et al. [21] (for non-athletes, mean HR for a 6-min normal cycling was 93.2 bpm, or 47% HR_max_, and for a 6-min intensive cycling was 131.6 bpm, or 66% HR_max_).

Based on health and ethical considerations, we also informed subjects before the program that if they experienced any progressively increasing chest pain, strong shortness of breath, strong feelings of fatigue, dizziness, vomiting, headache, stagger, or lower limb pain while exercising, they should inform the experimenter immediately. In these cases, the experimenter would immediately end the 10-min physical test. Furthermore, the physical test would also be terminated if the experimenter noticed any cyanosis, pallor of the face, cold sweat in the subject. As a result, four subjects reported strong feelings of fatigue during the 7–10 min of the physical test without any other subjective or objective symptoms. Therefore, the physical test was immediately terminated for these subjects, and all of them performed a cooldown for the rest of the intervention without reporting any other symptoms or request to quit the cooldown or withdraw from the experiment. Given there was no difference in the mean and maximal HR and self-reported mood after the intervention between these four subjects and the remaining 16 subjects in the exercise group, we included all 20 subjects in our final analysis.

For the control intervention, following Chang and Etnier [38], we asked subjects to read materials on the association between physical exercise and brain functions at a self-selected pace. The content of the materials was irrelevant to the creativity test and considered mood-neutral. Through pilot testing with volunteers, who had similar backgrounds to our subjects, we adjusted the amount of the materials to take roughly 15 min for most subjects to read. In the case of fast-readers, we prepared another set of materials on the same topic but with different contents.

During both interventions, subjects’ heart rate was monitored with an Apple Watch Series 4 (Apple Inc., California, United States), which has been shown to have high accuracy [39]. Unfortunately, due to an initial setting problem, data of the first minute in the exercise group where subjects stayed seated quietly on the exercise bike were not recorded. That is, for the exercise group, we had only HR data after they started to pedal. Nevertheless, since the HR in the first minute is expected to be similar between the exercise and control group, we do not consider this recording problem a serious issue and, therefore, used HR across the 14 min as the data for the exercise group.

### 2.4. Divergent Thinking

We used the AUT for the measurement of divergent thinking. Subjects were presented three common objects and asked to write down as many as possible uncommon, original, and unique uses of those objects in 4 min on A4 size blank paper. We prepared two sets of objects, and for each subject, one set was randomly selected for pre-test (before the intervention) and the other post-test (after the intervention). For set A, “brick”, “empty can”, and “umbrella”were used; for set B, “pencil”, “tissue box”, and “newspaper” were used.

For the scoring of the AUT, we used fluency, flexibility, and originality as indicators of creativity [27,28]. Fluency was defined as the number of generated uses. Flexibility was the number of different conceptual categories the alternative uses are from. Originality refers to the rareness of generated uses and was defined here based on the conceptual category of the uses, rather than the uses, per se. Only if one single subject gave use(s) from a specific conceptual category, the category counted as original; if two or more subjects gave use(s) from the same conceptual category, the category did not count as original for both or all of them. For instance, given “brick”, “road mark” belongs to one category, while “paperweight” and “stone weight” belong to another. If one subject gave “paperweight” and the other subject “stone weight”, the response counted as original for neither of them. A primary coder scored all responses, and a secondary coder scored responses for a randomly selected object. The two coders reached an agreement of Cohen’s κ = 0.936 for flexibility and Cohen’s κ = 0.706 for originality, which is considered substantial or almost perfect [40].

### 2.5. Convergent Thinking

We used the matchstick arithmetic problems developed by Knoblich et al. [41] to evaluate insight problem-solving or convergent thinking in our pre-test. A matchstick arithmetic problem consists of a false equation written with Roman numerals, for example, II = III + I. Subjects were required to move a single stick to transform the equation into a correct one. In the above example, the correct response is to move one stick from “III” on the right side to “II” on the left side. Four different classes of problems can be identified based on the kind of move, including moving a matchstick from a numeral to another numeral (type A), moving a matchstick from an operator sign to another operator sign or numeral (type B), rotating the vertical matchstick of the plus sign to form an equal sign (type C), and sliding one of the matchsticks from the symbol X to form V (type D).

Based on data reported by Knoblich et al. [41] and our pilot testing, we selected six problems and set 12 min as the time limit for solving these problems in our pre-test. The six problems included two problems from type A and B each and one problem from type C and D each. We expected that a total of six problems was adequate such that subjects would not be overwhelmed while at the same time most of them would not be able to solve all the problems. Therefore, this allowed us to present the unsolved problems to the subjects again at post-test to investigate whether exercise could help them gain insights on these previously unsolved problems. In the post-test, subjects were given three minutes to solve the unsolved or incorrectly solved problems. Those who correctly solved all pre-test problems were presented three new problems to work on at the post-test.

Before solving these problems in the pre-test, all subjects first went through a training session to ensure that all were familiar with Roman numerals. They were instructed to study a correspondence table between modern numerals (1–15) and Roman numerals (I through XV) until they passed two tests: in the first test, they were presented all the fifteen modern numerals in random order and asked to write down the corresponding Roman numerals; in the second test, vice versa, they were presented with all the Roman numerals and asked to write down the corresponding modern numerals.

For convergent thinking at post-test, we also included another set of creative problem-solving puzzles that require visuospatial and logical reasoning [42]. Tago [42] is a selected collection of creative puzzles written by the Japanese educator Akira Tago, from his series *atama no taiso* or Mental Gymnastics first released in 1966. The puzzles in the series have been considered a valid measure of creativity in the Japanese culture and used to evaluate creativity in scientific studies by Japanese researchers [43]. Based on our pilot testing, we selected three puzzles for our test here. As an example, the first puzzle had the background stating that three friends were about to eat three pieces of isosceles triangle-shaped cakes when one more friend joined them; the question was, what is the minimum number of times required for cutting these three pieces of cakes into four equal parts, in order for four of them to eat. Subjects were encouraged not to give up and to do their best to solve all the puzzles. If subjects finished answering all the puzzles before the time limit (i.e., 9 min), they were given four more puzzles to solve, the results of which were not analyzed here.

Therefore, for convergent thinking, we created two measures for data analysis, one was a creative problem-solving (CPS) score consisted of matchstick arithmetic problems at pre-test and creative problem-solving puzzles at post-test; the other was a re-test score on the matchstick arithmetic problems (or matchstick re-test) obtained at post-test only.

After subjects finished all the above convergent thinking tests, they filled out a survey asking whether they have seen any of the tests presented. Two subjects (both from the exercise group) had seen exactly the same creative problem-solving puzzle previously (the second and third puzzle) and, therefore, were excluded from relevant data analysis (where n = 18 for the exercise group).

### 2.6. Statistical Analysis

The statistical analysis was conducted with IBM SPSS Statistics 26.0. The normality of the data was checked using the Shapiro–Wilk test. Student’s *t*-tests or Mann–Whitney U tests were used for comparing between-group differences, while repeated measures ANOVAs were used for comparing between-group pre-test–post-test effects, with group (control vs. exercise) as between-group factor and time (pre-test vs. post-test) as the within-subjects factor. Due to an age difference between the two groups, age was included as a covariate in one-way ANCOVAs and repeated measures ANOVAs. Pearson’s or Spearman correlation analysis was used to examining the association between mood and creative thinking measures. Median analysis was conducted with Mplus 8.0 (Muthén and Muthén, 2012). Effect size (Cohen’s d) was calculated using G*Power Version 3.1.9.6 [44]. A significance level of *p* < 0.05 was used.

## 3. Results

### 3.1. Heart Rate and Mood Measures

**Figure 2:**
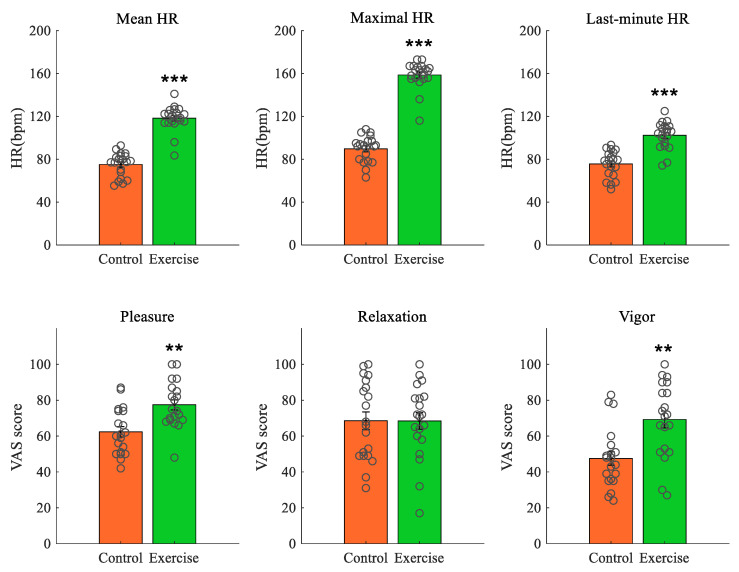
Intervention effect on heart rate (HR) and mood. **Upper** panels: HR; **lower** panels: mood. ** *p* < 0.01, *** *p* < 0.001 compared to control. Data shown as means ± SE, circles represent individual responses.

As plotted in Figure 2, compared to the control group, subjects in the exercise group had significantly higher mean heart rate (*U* = 4.000, *p* = 0.000, *d* = 3.72) and maximal heart rate (*U* = 0.000, *p* = 0.000, *d* = 5.47) during the intervention, and higher heart rate in the last minute of the intervention (*t*(38) = 6.766, *p* = 0.000, *d* = 2.14). Subjects in the exercise group also reported higher feelings of pleasure (*t*(38) = 3.707, *p* = 0.001, *d* = 1.17) and vigor (*t*(38) = 3.625, *p* = 0.001, *d* = 1.15), but not relaxation (*t*(38) = 0.015, *p* = 0.988). All these between-group differences remained significant after incorporating age as a covariate (one-way ANCOVAs). Given that the mean heart rate was equivalent to 60.2% age-estimated HR_max_ [31,32], our exercise intervention was considered primarily light in intensity, despite the fact that the maximal heart rate here had reached 80.8% age-estimated HR_max_ (considered vigorous in intensity). Furthermore, since the two groups did not differ in their positive and negative affect at baseline, the above results suggest that exercise increased subjects’ feelings of pleasure and vigor.

### 3.2. Divergent Thinking

**Figure 3:**
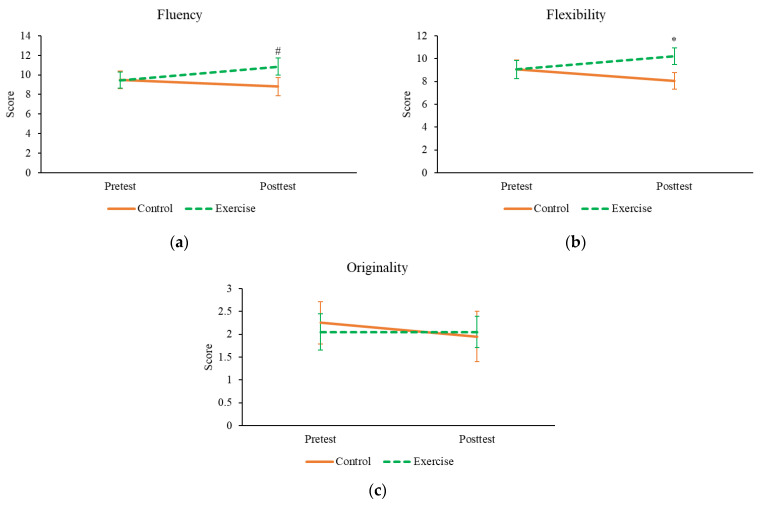
Exercise effect on divergent thinking (AUT). (**a**), fluency; (**b**), flexibility; (**c**), originality. * *p* < 0.05, indicating a significant effect of group × time interaction; #, *p* = 0.066 for the group × time interaction. Data shown as means ± SE.

The between-group pre-test–post-test comparisons of the AUT results are shown in Figure 3 and Table S2. There was a significant effect of group × time interaction on flexibility (F_(1,38)_ = 5.158, *p* = 0.029, *d* = 0.37) and a trend towards significance on fluency (F_(1,38)_ = 3.588, *p* = 0.066, *d* = 0.31). Similar results were obtained after, including age as a covariate (F_(1,37)_ = 4.898, *p* = 0.033 for flexibility, F_(1,37)_ = 3.866, *p* = 0.057 for fluency). As shown in Figure 3, exercise increased flexibility and fluency (with a trend) at post-test, without affecting originality.

### 3.3. Convergent Thinking

**Figure 4:**
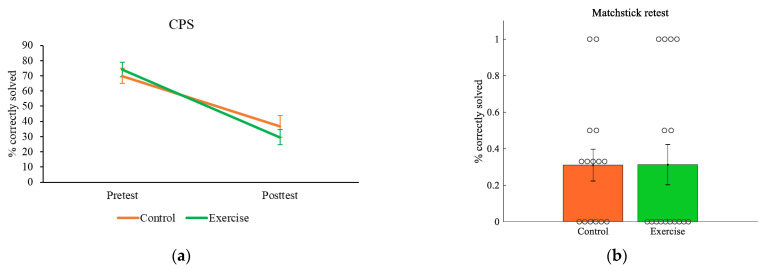
Exercise effect on convergent thinking. (**a**), CPS; (**b**), matchstick re-test. Data shown as means ± SE, circles represent individual responses.

The comparisons of the convergent thinking scores are shown in Figure 4 and Table S2. No significant effect of group × time interaction was obtained for the CPS score, although a significant effect of time (F_(1,36)_ = 57.134, *p* = 000, *d* = 1.26) indicating that subjects obtained significantly lower scores at post-test compared to pre-test was observed (Figure 4a). This difference, however, became nonsignificant after controlling age (Table S2). There was also no difference between the two groups in the score of the matchstick re-test (Figure 4b), which remained unchanged after controlling age.

### 3.4. Mood Effect

To explore whether mood regulated the exercise effect on divergent and convergent thinking, we conducted a correlation analysis for each group between creativity scores at post-test and subjects’ self-reported mood following the intervention. As shown in Table S3 and Figure S1, neither pleasure nor vigor was associated with AUT measures in the control or exercise group (all *p* > 0.10), although relaxation was positively associated with AUT measures in the control group (r/rho = 0.511–0.572, all *p* < 0.05). Whereas exercise boosted fluency and flexibility and increased pleasure and vigor, there were no correlations between these mood and creativity measures.

Based on the literature review mentioned in the Introduction, we further tested the hypothesis that exercise improves fluency and flexibility through its effect on mood, namely pleasure and vigor, using mediation analysis. Specifically, we tested three models for each outcome variable (i.e., fluency and flexibility): In the first model, pleasure and vigor concurrently mediate the effect of exercise, while in the second and third models, pleasure or vigor alone mediates the effect of exercise. Contrary to our hypothesis, none of the models indicated a significant indirect mediation effect (all *p* > 0.10; Table S4).

**Figure 5:**
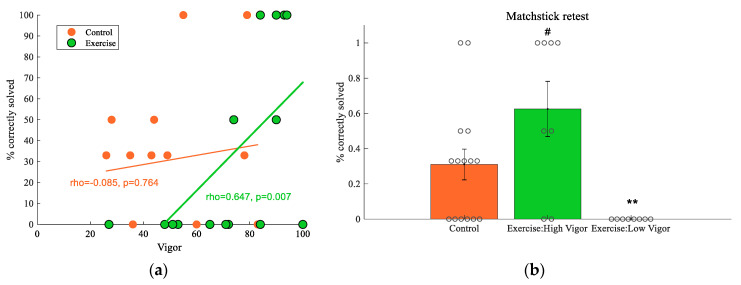
Exercise effect on matchstick re-test affected by vigor. (**a**) Scatterplot of the association (with a regression line) between post-intervention vigor and matchstick re-test score in each group; (**b**) comparison of matchstick re-test score between control and the two exercise groups (created based on a median split of vigor). **, *p* < 0.01; # *p* = 0.081, compared to control. Data shown as means ± SE, circles represent individual responses.

For convergent thinking, notably, there was a positive association between matchstick re-test score and pleasure and vigor in the exercise group only (Table S3, Figure 5a). Subjects reporting high feelings of pleasure or vigor after exercise showed better performance on the matchstick re-test. These correlations (for pleasure, rho = 0.503, *p* = 0.047; for vigor, rho = 0.647, *p* = 0.007) are considered moderate to moderately high in magnitude [45]. When we divided the exercise group into low and high vigor groups based on a median split, compared to the control group, subjects in the high vigor group (Exercise: High Vigor) tended to have a higher score on the matchstick re-test (*U* = 34.00, *p* = 0.081, *d* = 0.83, Figure 5b), while those in the low vigor group (Exercise: Low Vigor) had a significantly lower score on the matchstick re-test (*U* = 24.00, *p* = 0.008, *d* = −1.13). That is, compared to the control group, subjects reporting high vigor after exercise tended to solve more matchstick problems that were unsolved before the intervention. On the other hand, subjects reporting low vigor became even less capable of solving the previously unsolved problems. However, there were no such differences when we conducted a similar analysis with pleasure.

We also conducted another analysis to test whether subjects’ self-reported vigor post-exercise was associated with their physical fitness and exercise habits, based on the assumption that subjects with a low level of physical fitness and habitual physical activity may be more easily exhausted. A comparison of the low to the high vigor group indicated that the low group tended to have lower grip strength (*t*(14) = −1.948, *p* = 0.072, *d* = 0.97) and conduct less physical activity in the past seven days (total METS, *t*(10) = −1.895, *p* = 0.087, *d* = 0.95).

## 4. Discussion

In the present study, we found an exercise program that enhanced subjects’ self-reported mood (in terms of pleasure and vigor) improved the flexibility and fluency of divergent thinking. However, there was no correlation between flexibility and fluency and subjects’ self-reported pleasure and vigor post-exercise, and the creativity effect of exercise was not mediated by post-exercise self-reported pleasure and vigor. In contrast, the exercise effect on convergent thinking depended on subjects’ mood after exercise: subjects reporting high vigor tended to solve more insight problems unsolved previously, while those reporting low vigor became less capable of solving previously unsolved problems.

We used a built-in automatic physical test program for the exercise intervention, the intensity of which was considered falling between normal walking or cycling and cycling with maximal effort (as employed by Colzato et al. [21]). Whereas cycling with maximal effort impaired flexibility of divergent thinking in Colzato et al. [21], here our exercise intervention improved flexibility. This may partially support the hypothesis we speculated in the Introduction that exercises at low-to-moderate intensity might be better able to enhance divergent thinking than exercise at too low or too high an intensity ([21,22,23]). The hypothesis, however, still requires further confirmation by future studies with more homogenous and accurately prescribed intensities.

Our findings only partially confirm our prediction that the creativity effect of exercise may depend on its mood effect since the effect of exercise on convergent but not divergent thinking is mood-dependent. Our speculation that Colzato et al. [21] and Frith and Loprinzi [23] failed to find the divergent thinking-enhancing effect because they used exercise interventions that did not change subjects’ mood is, therefore, not supported. Our prediction was considered possible because it was consistent with two lines of neurobiological findings: first, physical exercise improves mood at least partly through increasing neurotransmitters, including serotonin, dopamine, and endocannabinoids (e.g., [46,47,48,49]; for a review, see [7]); and second, these neurotransmitters play a critical role in cognition [50,51,52] and are also involved in the cognitive-enhancing effect of exercise [7]. Our results, however, showed that, while the effect of exercise on convergent thinking depends on mood, its effect on divergent thinking does not. This raises the possibility that convergent and divergent thinking might share distinct neurobiological mechanisms. Our findings are consistent with Steinberg et al. [19], who reported that the divergent thinking enhancing effect of exercise was independent of mood, although a Likert scale rather than visual analog scale [53] was used in that study. Future studies are required to test the possibilities that we suggested and uncover the underlying explanations of why the effect of exercise on convergent but not divergent thinking is mood-dependent.

For the first time, we showed that the effect of exercise on convergent thinking depends on post-exercise mood such that high vigor may enhance, while low vigor impairs creative problem-solving. This finding is in line with studies showing that exercise without causing changes in mood failed to improve convergent thinking using the RAT and CRA [21,23]. Previous research has indicated a critical role of serotonin, dopamine, and endocannabinoids in feelings of vigor [54,55] and behavioral indicators of vigor (e.g., performance or response vigor, [56,57]). Generally, exercise at an adequate intensity enhances vigor, while that at too high an intensity causes central fatigue and reduces vigor, likely through the interactions of serotonin and dopamine in the brain [58]. As such, it is possible that subjects reporting low vigor after exercise in our study may have been exhausted, which impaired their cognitive functions. Indeed, our analysis showed that these subjects tended to have lower grip strength, an important index of physical fitness [32], and conduct less physical activity in the past seven days. Considering that our exercise program reached vigorous intensity in the last three minutes, these subjects might have been more easily exhausted. This speculation is in line with Colzato et al.’s [21] findings that cycling with maximal effort (moderate intensity) impaired RAT performance in non-athletes but tended to improve RAT performance in athletes. It is also consistent with a recent report that jogging acutely enhanced visual attentional control, the effect of which was fully mediated by feelings of energy [59]. Future studies must further investigate these possibilities and clarify the association between post-exercise vigor and convergent thinking.

Our findings suggest that engaging in an acute bout of aerobic exercise enhances divergent thinking, and when it improves vigor, convergent thinking may also be enhanced. Therefore, aerobic exercise may be considered a simple but useful strategy for improving these cognitive functions. It may also have important therapeutic potential in psychiatric contexts since many psychiatric conditions, such as depression, have been associated with impaired divergent thinking and problem-solving abilities [60,61,62,63,64].

Several important limitations of our study should be noted. First, we used a built-in automatic physical test program for the exercise intervention and provided a post hoc estimation of the exercise intensity based on age-estimated maximal heart rate. A preferred way to account for the individual differences in aerobic capacity is to prescribe a homogenous intensity by referring to a percentage of the aerobic capacity reserve [8,31]. Neither did we have the participants rate their level of perceived exertion during the exercise intervention, which provides a subjective evaluation of the intensity [8]. A second limitation of the study is that we used a between-subjects pre-test–post-test design. As already stated in Materials and Methods, a within-subjects crossover pre-test–post-test design may be more rigorous [8]. However, the nature of the creative thinking tests hindered us from using this design. We hope more insight problems and convergent thinking tests will be developed and validated in the near future so that we will be able to use more rigorous designs. A third limitation is actually related to the insight problems we used to measure convergent thinking. As shown in Figure 4A, subjects on average performed poorly on the CPS at post-test. A closer look at subjects’ responses indicated that 30 subjects (75%) failed to solve any puzzle or correctly solved merely one puzzle. Due to this high level of difficulty, a floor effect may have occurred that hindered us from detecting the specific, creativity-enhancing effect of exercise. Future research needs to use convergent thinking tests with an appropriate level of difficulty to confirm our findings.

Lastly, our sample size was relatively small, and although the data of AUT-originality and matchstick re-test were not normally distributed, we still used two-way ANOVA and one-way ANCOVA for our main analysis. These tests were considered rather robust, and few nonparametric tests existed for these situations [65]. Future studies with large sample sizes, more robust statistical tests and appropriate statistical powers are required to confirm the influence of mood on the creativity effects we reported here. We hope the current exploratory study may provide useful preliminary findings for more rigorous studies in the future.

## 5. Conclusions

In a randomized controlled trial with a between-subjects pre-test–post-test comparison design, we found that a 15-min exercise program improved divergent thinking in terms of fluency and flexibility, and the effect did not depend on subjects’ self-reported mood after exercise. The exercise program, however, affected convergent thinking in a mood-dependent manner such that subjects reporting high vigor tended to solve more insight problems that were unsolved previously. In contrast, those reporting low vigor became less capable of solving previously unsolved problems. Although our sample size was relatively small, these findings suggest the possibility that engaging in a bout of mood, particularly vigor-enhancing aerobic exercise, may be considered a simple but useful strategy for gaining insights into previously unsolved problems. Future more rigorous studies are required to replicate our findings.

## Data Availability

The data that support the findings of this study are available from the corresponding author upon reasonable request.

## Supplementary Materials

The following are available online at https://www.mdpi.com/article/10.3390/brainsci11050546/s1, Table S1: Summary of previous studies on the after-effects of acute physical exercise on creative thinking; Table S2: A summary of main statistical results: repeated measures ANOVA; Table S3: Correlation between self-reported mood and divergent and convergent thinking at post-test; Table S4: Mediation models and results; Figure S1: Scatterplot (with a regression line) of correlation between mood and creative thinking measures at post-test.
