# Supplementary material for: The Effect of Acute Aerobic Exercise on Divergent and Convergent Thinking and Its Influence by Mood
Source: Brain Sci. 2021 Apr 27;11(5):546. doi: 10.3390/brainsci11050546 (PMC8145661; doi:10.3390/brainsci11050546)
Supplement: Supplementary file 1 [file brainsci-11-00546-s001.zip › brainsci-1178575-supplementary.pdf]

Table S1. Summary of previous studies on the after effects of acute physical exercise on creative thinking [a].

	**Steinberg et al 1997 [19]**	**Netz et al., 2007 [20]**	**Colzato et al 2013 [21]**	**Oppezzo & Schwartz 2014 [22]**	**Frith & Loprinzi 2018 [23]**	**Román et al 2018 [24]**
Subjects	Mainly college students and staffs: median age range 20-29 (n=63)	Adults aged 50-64 years (n=58)	Non-athletes [b]: mean age 20.7 years (n=48)<br>Athletes [c]: mean age 20.6 years (n=48)	College students (n=48 for treadmill walking & n=40 for outdoor walking)	College students: age 23.1 ±3.39 years (n=32)	Children: age 9.84 ±1.12 years (n=96)
Design	Within-subjects crossover posttest: different day	Between-subjects pretest posttest	Within-subjects crossover posttest: same day	Between-subjects posttest [d]	Within-subjects crossover posttest: different day	Between-subjects pretest posttest
Exercise intervention	Aerobic workout or dance (17-22 min)	Treadmill walking (44 min): 60-70% HRR	Normal cycling (6 min): mean HR 93.2; 94.4 bpm<br>Intensive cycling (6 min): cycle with maximal effort; mean HR 131.6; 126.1 bpm	Walking (4 min): treadmill or outdoor	Treadmill walking (15 min): HR at the end of walking 115.1 ±20.3 bpm	Physical education class (45 min): aerobic games
Control intervention	Neutral video watching	Movie-watching (on the topic of nature)	Sitting (6 min): on an ergometer; mean HR 75.5; 77.0 bpm	Sitting	Sitting (15 min): HR 75.3 ±12.5 bpm<br>Listening to self-selected music (15 min): HR 75.1 ±12.5 bpm	Sedentary class (45 min): e.g., mathematics & language
Timing of creativity test	Following a mood test (< 2-3 min)	5 min later	Following a HR, BP, and mood test (< 6 min)	Immediately	1 min rest & mood test	20 min later
Divergent thinking: AUT	Fluency: *ns*<br>Flexibility: ↑<br>Originality: *ns*	Fluency: ↑	Fluency: *ns*<br>Flexibility: Intensive ↓<br>Originality: *ns*<br>Elaboration: *ns*	Originality: ↑	Fluency: *ns*<br>Flexibility: *ns*<br>Originality: *ns*<br>Elaboration: *ns*	Adapted version<br>Fluency: ↑<br>Flexibility: ↑<br>Originality: *ns* v.s. Control (p=0.062)<br>↑ v.s. Pretest

Divergent thinking: other tests	-	-	-		-	Realistic presented problem: *ns*<br>Realistic problem generation: *ns*	Graphical creativity [e] ↑
Convergent thinking: RAT or CRA	-	-	Intensive: ↓<br>Normal: *ns*	Intensive: ↑ (*p*=0.095)<br>Normal: ↑ (*p*=0.072)	*ns*	*ns*	-
Other findings	Positive mood ↑<br>Negative mood ↓<br>Exercise effect was independent of mood	-	Arousal: Intensive ↑<br>Mood: *ns*		-	Happiness: *ns*<br>Arousal: Exercise > Seated control	-

AUT: Alternate Uses Test; **RAT**: Remote Associates Test; **CRA**: Compound Remote Associates Test; **HR**: heart rate; **HRR**: Heart rate reserve; **BP**: blood pressure; ***ns***: non-significant. [a] **Literature search strategy**: *database*: Pubmed; *search field*: Title/Abstract; *search terms*: (exercise OR physical activity OR walking OR running OR cycling) AND (acute OR bout) AND (creativity OR divergent thinking OR convergent thinking OR Alternate Uses OR Guilford OR Torrance OR remote associates test OR cognitive flexibility); *last search and confirmation date*: 2020/12; a cross-reference search was also conducted; we also identified one study with Hatha yoga combining yoga postures and meditation etc. (Bollimbala, A., James, P. S., & Ganguli, S. (2020). The effect of Hatha yoga intervention on students' creative ability. Acta Psychologica, 209, 103121.), which was not included in the review here. [b] non-athletes: subjects who exercised less than once a week during the past two years; [c] athletes: subjects who exercised at least three times a week during the past two years; [d] The comparison between Walk-Sit v.s. Sit-Sit in experiment 2 & 3 is reported here; [e] Graphical creativity: in this task, subjects were asked to complete unfinished drawings with high originality and think of an interesting title for each drawing.

Table S2. A summary of main statistical results: repeated measures ANOVA.

		Divergent thinking			Convergent thinking	
		**AUT-Fluency**	**AUT-Flexibility**	**AUT-Originality**	**CPS**	**Matchstick retest [a]**
Group		$F_{(1,38)}$=0.808, p=0.374	$F_{(1,38)}$=1.210, p=0.278	$F_{(1,38)}$=0.009, p=0.925	$F_{(1,36)}$=0.059, p=809	$F_{(1,29)}$=0.000, p=0.992
Time		$F_{(1,38)}$=0.399, p=0.532	$F_{(1,38)}$=0.025, p=0.875	$F_{(1,38)}$=0.201, p=0.657	**$F_{(1,36)}$=57.134, p=0.000**	-
Group*time interaction		**$F_{(1,38)}$=3.588, p=0.066**	**$F_{(1,38)}$=5.158, p=0.029**	$F_{(1,38)}$=0.201, p=0.657	$F_{(1,36)}$=1.166, p=287	-
Age as covariate	Group	$F_{(1,37)}$=0.997, p=0.324	$F_{(1,37)}$=1.267, p=0.268	$F_{(1,37)}$=0.098, p=0.756	$F_{(1,35)}$=0.138, p=0.713	$F_{(1,28)}$=0.595, p=0.447
	Time	$F_{(1,37)}$=0.401, p=0.531	$F_{(1,37)}$=0.085, p=0.772	$F_{(1,37)}$=0.530, p=0.471	$F_{(1,35)}$=0.030, p=0.865	-
	Group*time interaction	**$F_{(1,37)}$=3.866, p=0.057**	**$F_{(1,37)}$=4.898, p=0.033**	$F_{(1,37)}$=0.038, p=0.846	$F_{(1,35)}$=0.761, p=0.389	-

AUT: Alternate Uses Test; CPS: creative problem-solving (matchstick arithmetic problems at pretest, creative problem-solving puzzles at posttest). [a] Subjects that correctly solved all matchstick puzzles at pretest were removed from this analysis; one-way ANOVA and ANCOVA were used. Although the data of AUT-Originality and matchstick retest were not normally distributed, we still used the two-way ANOVA and one-way ANCOVA because these tests were rather robust and few nonparametric tests exist for these situations (Chapter 10 and 12, Jerrold H. Zar 2010 Biostatistical Analysis, 5th Edition. Upper Saddle River, N.J.: Prentice-Hall/Pearson).

Table S3. Correlation between self-reported mood and divergent and convergent thinking at posttest.

	Divergent thinking			Convergent thinking	
	**AUT-Fluency**	**AUT-Flexibility**	**AUT-Originality**	**CPS**	**Matchstick retest**
**Control**					
Pleasure	r=0.369, p=0.110	r=0.300, p=0.199	rho=0.208, p=0.380	r=-0.019, p=0.646	rho=-0.181, p=0.519
Relaxation	**r=0.572, p=0.008**	**r=0.530, p=0.016**	**rho=0.511, p=0.021**	r=-0.064, p=0.789	rho=-0.242, p=0.385
Vigor	r=0.128, p=0.591	r=0.127, p=0.594	rho=0.376, p=0.102	r=-0.264, p=0.260	rho=-0.085, p=0.764
**Exercise**					
Pleasure	r=0.191, p=0.419	r=0.287, p=0.220	rho=-0.043, p=0.858	r=0.337, p=0.172	**rho=0.503, p=0.047**
Relaxation	r=0.280, p=0.231	r=0.305, p=0.191	rho=-0.095, p=0.689	r=-0.224, p=0.372	rho=0.149, p=0.583
Vigor	r=0.103, p=0.665	r=0.141, p=0.553	rho=-0.258, p=0.272	r=0.168, p=0.504	**rho=0.647, p=0.007**

For Control, n=20 for AUT, n=20 for CPS, n=15 for matchstick retest; For Exercise, n=20 for AUT, n=18 for CPS, n=16 for matchstick retest

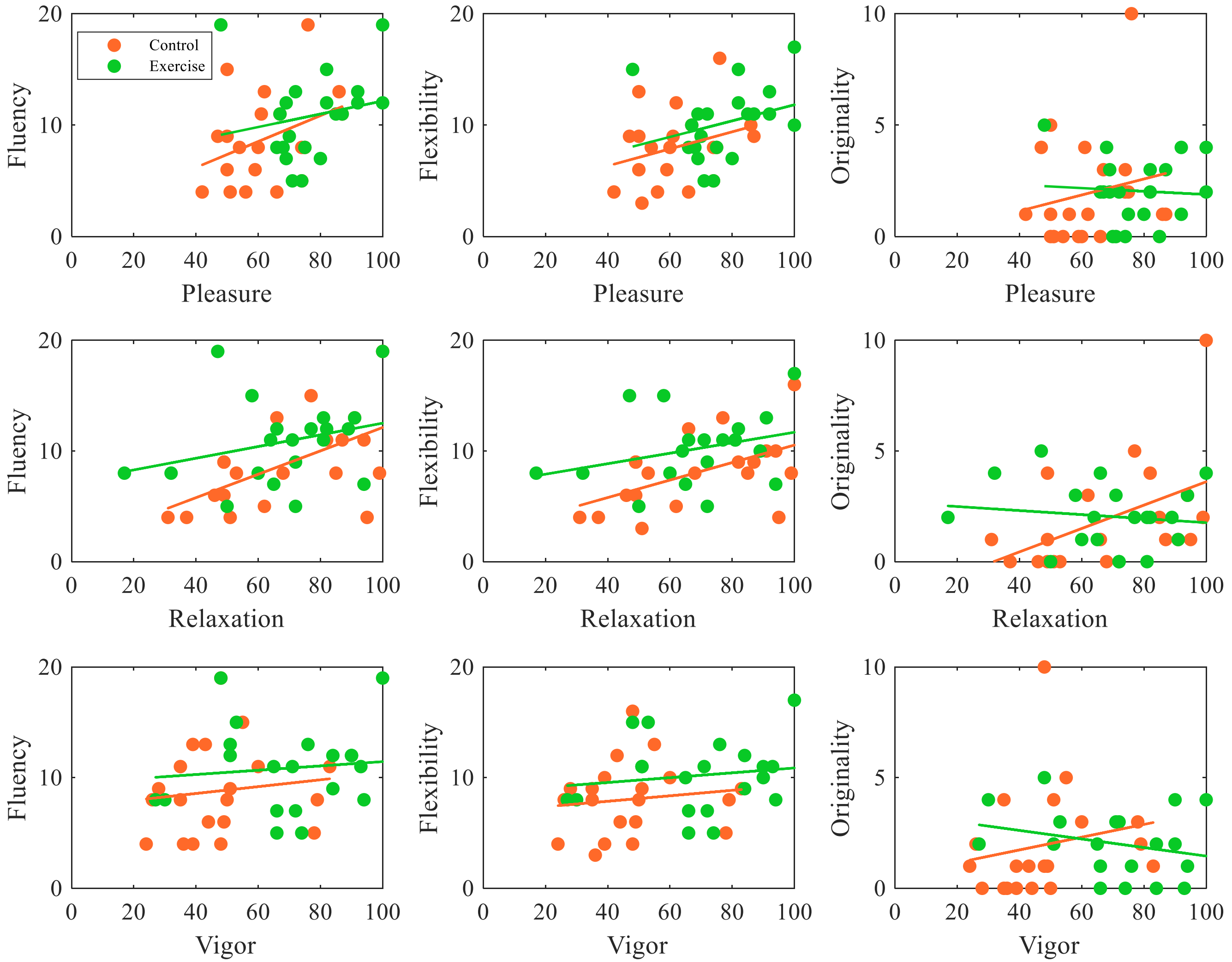

Figure S1. Scatterplot (with a regression line) of correlation between mood and creative thinking measures at posttest. Correlation coefficients are reported in Table S3.

Table S4. Mediation models and results.

	Outcome variable	Mediator(s)	Indirect effect(s)
Model 1		Pleasure & Vigor	p=0.277, p=0.692
Model 2	AUT-Fluency	Pleasure	p=0.265
Model 3		Vigor	p=0.506
Model 4		Pleasure & Vigor	p=0.201, p=0.762
Model 5	AUT-Flexibility	Pleasure	p=0.191
Model 6		Vigor	p=0.413
